# Trends in the use of validated claims-based algorithms in Japanese post-marketing database studies

**DOI:** 10.3389/fphar.2025.1642490

**Published:** 2025-09-02

**Authors:** Chieko Ishiguro, Takahiro Nonaka

**Affiliations:** ^1^ Laboratory of Clinical Epidemiology, Department of Data Science, Center for Clinical Sciences, Japan Institute for Health Security, Tokyo, Japan; ^2^ Department of Clinical Research Governance, The University of Tokyo Hospital, Graduate School of Medicine, The University of Tokyo, Tokyo, Japan

**Keywords:** real world evidence, validity, claims-based algorithm, database, post-marketing study, regulatory framework, risk management plan, pharmacovigilance

## Abstract

**Background:**

In pharmacoepidemiological research, misclassification is a concern with claims-based algorithms (also called computable phenotypes). Validating them is crucial, particularly within regulatory settings. However, the extent of their application remains unclear globally.

**Objectives:**

This study aimed to investigate the frequency and trends of validated claims-based algorithms use in post-marketing database studies.

**Method:**

We reviewed all Japanese risk management plans published until January 2023, identifying four issue types [Effectiveness Issues (EI), Important Identified Risks (IIR), Important Potential Risks (IPR), and Important Missing Information (IMI)] that were planned to use a claims-based algorithm in post-marketing database studies. We then calculated the proportion of issues intending to use a validated claims-based algorithm, and performed subgroup analyses by issue type.

**Results:**

Of 68 issues (3 EI, 47 IIR, 13 IPR, 5 IMI), 15 (22.1%) planned to use a validated algorithm, all for outcome definitions; 10 to conduct new validation studies and 5 to refer to existing studies, including studies with high positive predictive value and sensitivity. Subgroup analyses by issue type showed that the proportions were 100% for EI, 17.0% for IIR, 30.8% for IPR, and 0% for IMI.

**Conclusion:**

Validated algorithm use was the highest for effectiveness issues but limited for safety, suggesting that results from these post-marketing database studies for safety issues may not provide sufficient evidence, highlighting the need to promote the use of validated claims-based algorithms. Future studies should use more recent data, compare the use of validated algorithms between Japan and other countries, and explore barriers to their adoption.

## 1 Introduction

Drug safety evaluation in the post-approval phase has advanced significantly over the past 2 decades. This is attributed to the emerging secondary use of administrative claims data or electronic health records for pharmacoepidemiology studies, mentioned as a safety monitoring method in the International Council for Harmonisation of Technical Requirements for Pharmaceuticals for Human Use E2E guidelines (International council for harmonisation of technical requirements for pharmaceuticals for human Use, 2024a). Pharmacoepidemiological studies using such databases have become widespread worldwide, leading to numerous safety-related regulatory actions (Prilla et al., 2024; Kajiyama et al., 2024; Maro et al., 2023). In addition, the publication of Step 2 of the ICH M14 guidelines has further focused on the utilization of healthcare databases for safety evaluation (International council for harmonisation of technical requirements for pharmaceuticals for human Use, 2024b).

Pharmacoepidemiological studies using a claims database commonly define study elements (target population, exposure, outcome, and covariates) via algorithms (Ehrenstein et al., 2024), also known as computable phenotypes (Rivera et al., 2025), which are constructed by combining various types of information, such as diagnostic codes, drug codes, and procedure codes. This is because these databases are created primarily for clinical and billing purposes and a lot of the information is not directly available for research. However, these algorithms may not identify the intended individuals, and misclassification arising during the creation of the analytical dataset can introduce systematic errors (information bias) into study findings.

Various international guidelines have been released concerning the validity of algorithms. Guidelines issued by regulatory agencies such as the Food and Drug Administration and the European Medicines Agency recommend the use of an optimal algorithm supported by validation studies to minimize misclassification (U.S. Food & Drug Administration, 2024; European Medicine Angecy, 2023). A white paper endorsed by the International Society for Pharmacoepidemiology has proposed a practical framework for researchers and regulators to plan and assess the fit-for-purpose of each study element (i.e., the required level of certainty for the algorithms depends on the study’s intent and specific context) in studies utilizing real-world data for regulatory decision-making (Cocoros et al., 2021). The HARPER guidelines, which promote transparency and reproducibility in observational studies using healthcare databases, recommend disclosing the validity of each algorithm used to define each study element (Wang et al., 2022).

In Japan, post-marketing database studies have been implemented as additional pharmacovigilance activities since the revised Good Post-Marketing Study Practice was implemented in 2018 (Ministry of Health, Labour and Welfare, 2017). The next year, the validation study guideline for post-marketing database studies was published by the Pharmaceuticals and Medical Devices Agency (PMDA). This guideline requires the use of a validated algorithm when the result from a post-marketing database study is considered the main evidence for a specific safety-related regulatory action (Pharmaceuticals and Medical Devices Agency, 2020). However, the criteria remain ambiguous, and the frequency and context of validated algorithm usage in post-marketing studies remain unknown globally. Understanding this frequency and context is essential for evaluating how guidelines are implemented in practice, clarifying which types of safety issues require validated algorithms in regulatory decision-making, and identifying potential gaps in Japan’s safety monitoring system. Therefore, we aimed to investigate the frequency and current trends of using validated algorithms in post-marketing database studies in Japan.

## 2 Methods

### 2.1 Data sources

We used information from the pharmacovigilance planning section of Japanese Risk Management Plans (J-RMPs) published on the PMDA website up to January 2023. This study obtained data on the latest and previous J-RMPs from the EPS Corporation (Tokyo, Japan), a company that has collected all J-RMPs published on the PMDA website since 2013 (EPS Corporation, 2024). J-RMPs for each new drug are developed by marketing authorization holders (MAHs) to comply with regulatory requirements, including plans for post-marketing studies addressing “effectiveness issues” or “safety issues” identified before approval. Safety issues listed in the safety specification section of J-RMPs are categorized into three types: “important identified risks,” defined as important adverse events whose association with the drug is shown based on sufficient evidence; “important potential risks,” usually defined as important adverse events whose association with the drug is suspected due to some factors, but is not sufficiently confirmed by clinical data, or drug-drug interactions suspected based on pharmacological mechanisms; and “important missing information,” usually comprising cases where sufficient critical information has not been obtained at the time of J-RMP development, thus lacking information to predict safety in the post-marketing phase of the drug. Important missing information usually includes specific populations excluded from clinical trials, despite the expected high frequency of drug use in these populations in real-world practice (Ministry of Health, Labour and Welfare, 2012).

### 2.2 Study design and study cohort

This cross-sectional descriptive study examined the effectiveness and safety issues planned in post-marketing database studies using claims databases in Japan. Issues considered in post-marketing database studies listed in all J-RMPs published up to January 2023 were identified as candidates for the study cohort. When multiple issues (e.g., important identified risk and important missing information) were covered within a single database study, each issue was counted separately. If multiple post-marketing database studies with different research questions were planned for a single issue, the issue was counted for each study. The following issues were excluded: 1) database selection in progress, 2) safety issues not listed in the safety specifications of the J-RMP, 3) issues with insufficiently documented definitions, and 4) issues defined using data other than claims data (administrative documents for medical expense reimbursement), such as laboratory test results data and registry data. Therefore, this study cohort only included issues assumed to be defined using claims-based algorithms.

### 2.3 Classifying algorithms as validated or non-validated

When post-marketing database studies require the use of validated algorithms as a regulatory condition, this requirement is generally documented in the pharmacovigilance planning section of the J-RMP. We investigated plans to use a validated algorithm for the definition of a study element in a post-marketing database study corresponding to a main concern in each issue. If no description indicated the use of a validated algorithm, or the use of such an algorithm was considered, these issues were treated as “planned to use a non-validated claims-based algorithm.” When the use of a validated algorithm was clearly described, these issues were treated as “planned to use a validated claims-based algorithm,” including cases referring to an existing validated algorithm and those involving a new validation study. An MAH may plan to use validated algorithms even without regulatory requirements; however, as these cases are not described in the J-RMP, they were considered outside the scope of this study.

### 2.4 Covariates

We considered the following covariates in our analysis: types of issue (effectiveness issues, important identified risks, important potential risks, and important missing information); study elements in post-marketing database studies corresponding to a main concern in each issue (outcome, population, and exposure); ICD-10 major classification of issues (only for outcomes); and fiscal year (April–March) when post-marketing database studies were added to J-RMPs.

### 2.5 Statistical analysis

We calculated the proportion of issues planning to use a validated claims-based algorithm, as defined via claims data. We conducted subgroup analyses according to the issue type, study element, fiscal year (FY 2017-18, FY 2019-20, FY 2021-22), and ICD-10 major classification of issues.

## 3 Results

**FIGURE 1:**
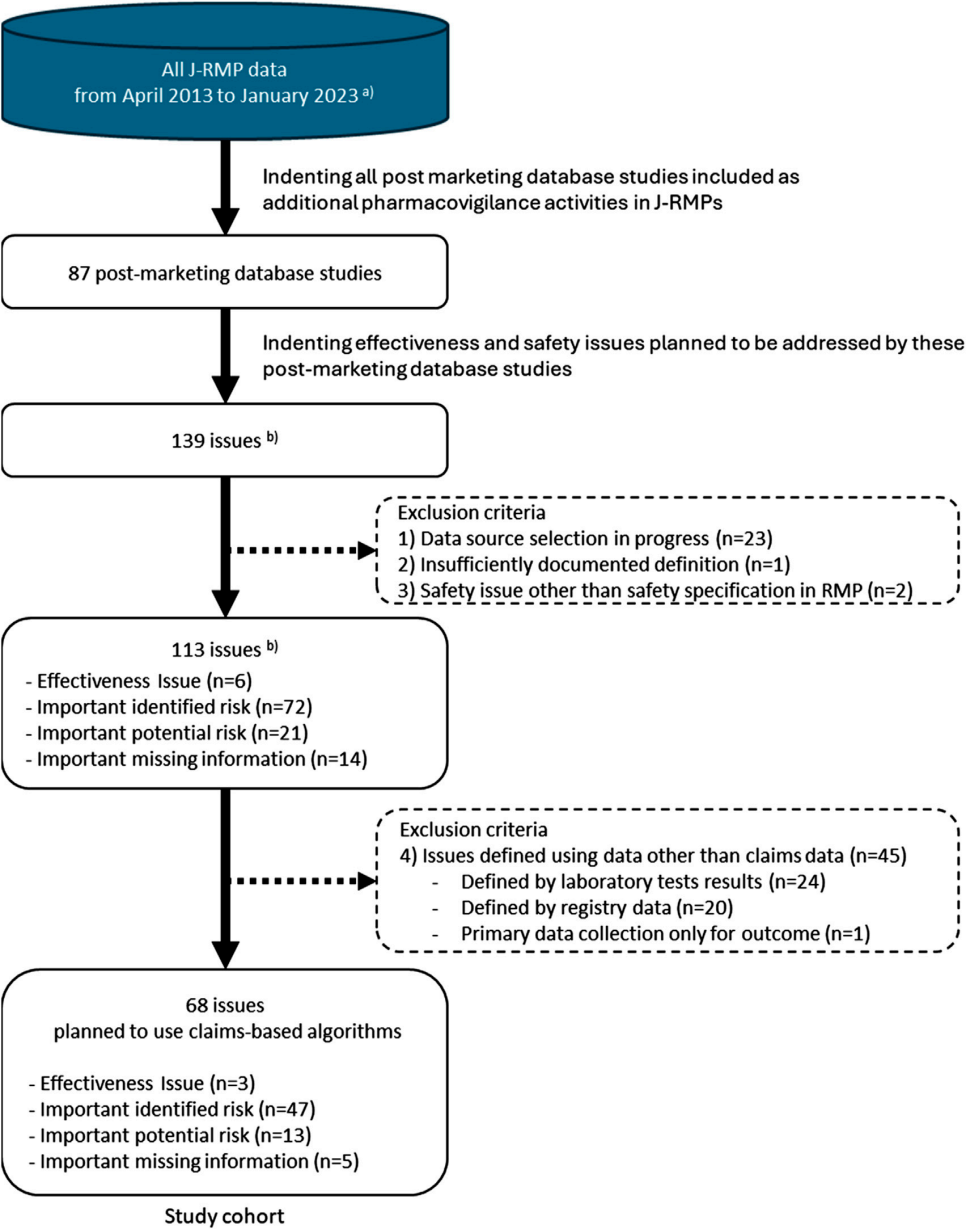
Study cohort selection. J- RMP: Japanese Risk Management Plan. **(a)** The database was maintained by the EPS corporation. **(b)** When different post-marketing database studies within a J-RMP assessed the same issue, the issue was counted for each study. When multiple issues (e.g., important identified risk and important missing information) were covered within a single database study, each issue was counted separately.

Eighty-seven post-marketing database studies covering 139 issues were included as candidates. We excluded 71 issues, including 23 under database selection, one with insufficiently documented definition, two that were not listed in the effectiveness or safety specifications of the J-RMP, and 45 defined using data other than claims data (24 laboratory test results, 20 registry data, and one primary data collection for outcome). The excluded issues are listed in Supplementary Tables S1, S2. The study cohort consisted of 68 issues defined using claims-based algorithms (Figure 1).

The basic characteristics of the 68 issues are shown in Table 1. Supplementary Table S3 shows the details of each issue. They comprised three effectiveness issues, 47 important identified risks, 13 important potential risks, and five cases of important missing information. The distribution by year was as follows: 31 issues in 2017–18, 18 issues in 2019–20, and 19 issues in 2021–22. Among the 47 important identified risks, 45 issues were related to outcomes (adverse events suspected to be causally related to the drugs), for which post-marketing database studies were planned to address the causal relationship between the drugs and adverse events. Among the 13 important potential risks, 11 issues were related to outcomes (adverse events with established causality), for which post-marketing studies were planned to obtain information that contributes to risk minimization, such as the frequency or severity under real-world use, or the identification of high-risk groups. All important missing information was related to exposure.

**TABLE 1:** Characteristics of safety issues and the frequency of use of validated claims-based algorithms.

	Total number of issues	Number of issues defined using validated claims-based algorithms	(%)
All issues	68	15^d^	22.1%
Subgroup			
Type of issue			
Effectiveness	3	3	100%
Important Identified Risks	47	8	17.0%
Important Potential Risks	13	4	30.8%
Important Missing Information	5	0	0%
Fiscal year^a^			
2017–2018	31	9	29.0%
2019–2020	18	4	22.2%
2021–2022	19	2	10.5%
Study element			
Outcome	59	15	25.4%
Population^b^	0	0	0%
Exposure^c^	9	0	0%
ICD-10 major classification of outcomes			
A-B: Certain infectious and parasitic diseases	10	3	30.0%
C: Neoplasms	5	2	40.0%
D: Diseases of the blood and blood-forming organs and certain disorders	5	2	40.0%
E: Endocrine, nutritional, and metabolic diseases	9	2	22.2%
G: Diseases of the nervous system	1	1	100%
I: Diseases of the circulatory system	18	3	16.7%
J: Diseases of the respiratory system	4	0	0%
K: Diseases of the digestive system	1	0	0%
M: Diseases of the musculoskeletal system and connective tissue	1	0	0%
N: Diseases of the genitourinary system	1	0	0%
Q: Congenital malformations, deformations, and chromosomal abnormalities	1	1	100%
T: Injury, poisoning, and certain other consequences of external causes	3	1	33.3%

^a^ 2017–2018: 2017 April - 2019, March 2019–2020: 2019 April - 2021, March 2021–2022: 2021 April −2023, January.

^b^ Patients with low body weight (1 issue), Patients with renal impairment (2 issues).

^c^ Drug interaction 4 (issues), drug switching (3 issues), long-term use (2 issues).

^d^ Including cases referring to an existing validated algorithm (10 issues) and those involving a new validation study (5 issues).

The frequencies of validated algorithms are shown in Table 1. Among the 68 issues, 15 (22.1%) planned to use validated claims-based algorithms. Subgroup analysis by issue type revealed that the highest frequency was 100% for effectiveness issues, followed by 30.8% for important potential risks, 17.0% for important identified risks, and 0% for important missing information. Subgroup analysis by fiscal year showed frequencies of 29.0% in 2017–18, 22.2% in 2019–20, and 10.5% in 2021–22. Subgroup analysis by study element showed frequency of 25.4% in outcome, 0% in other study elements. Subgroup analysis by disease class was limited by the small sample size, making it difficult to confirm trends. The 15 issues included the following outcomes: infectious diseases (3 issues), malignancies (2 issues), agranulocytosis/leukopenia (1 issue), myelosuppression (1 issue), hypoglycemia (1 issue), lactic acidosis (1 issue), peripheral and optic neuropathy (1 issue), heart failure/pulmonary edema (1 issue), stroke (1 issue), cardiovascular disease (1 issue), toxoplasmosis (1 issue), and shock/anaphylaxis/hypersensitivity/infusion reaction (1 issue). For 10 out of the 15 issues, new validation studies were planned; however, the details of their methodologies were unknown because the protocols had not been disclosed. For the remaining 5 issues, it was planned to refer to the results of existing validation studies, as detailed in Supplementary Table S4. Among these, three involved the PMDA, while the remaining two were conducted by a MAH. Except for one study for which details are unknown, these studies used medical chart review as the gold standard and reported both positive predictive value and sensitivity.

## 4 Discussion

We investigated the frequency and trends in the use of validated claims-based algorithms for effectiveness and safety issues planned for evaluation in post-marketing database studies using claims databases in regulatory settings. Our study revealed that the frequency of validated algorithm use varied across issue types; in particular, usage was very high for effectiveness evaluations, but much lower for safety evaluations. Furthermore, all validated algorithms were used exclusively for outcome definitions—although some issues involved defining populations or exposure status, no validated algorithms were used for these purposes. Subgroup analysis by fiscal year showed that the frequency of use decreased over time.

Validated algorithms were used most commonly for effectiveness issues, but there are only three effectiveness issues in this study and it is challenging to generalize these results. Given that post-marketing database studies as additional pharmacovigilance activities in J-RMPs could affect safety-related regulatory decision-making, the use of validated algorithms is always desirable. However, this study demonstrated a limited application of validated algorithms for addressing safety issues. The purpose of post-marketing database studies for safety issues may be either exploratory or confirmatory, which can influence whether a validated algorithm is used. In addition, the PMDA guideline requires a validated algorithm when the result from a post-marketing database study is considered the main evidence for a specific safety-related regulatory action (Pharmaceuticals and Medical Devices Agency, 2020). Further investigation, such as interviews with regulatory authorities and MAHs, will be necessary because it was not possible to determine whether the purpose was exploratory or confirmatory from publicly available information.

The validity of diagnosis codes in Japanese administrative claims data is generally low, so both the PMDA guideline for validation studies and the guideline issued by the Japan Society of Pharmacoepidemiology emphasize that, in database studies, particular attention should be paid to ensuring the validity of algorithms for outcome definitions (Pharmaceuticals and Medical Devices Agency, 2020; Iwagami et al., 2018). The latter guideline also highlights the importance of validating the algorithms used for study population definitions, especially when they are based on diagnosis codes. Conversely, prescription and dispensing data are typically standardized and electronically stored, which is generally considered to guarantee a certain level of validity (Iwagami et al., 2018) Our study found that validated claims-based algorithms were employed exclusively for outcome definitions, and, as far as we could confirm, validation studies planned to be referenced demonstrated high validity. In addition, non-validated definitions were planned for exposure-related issues, and all population-related issues were excluded from this study because laboratory values or registries defined these populations. These results were thought to align with the content of these guidelines.

The limited use of validated claims-based algorithms for safety issues may be attributable to various barriers to conducting validation studies in Japan. For example, links between insurers’ claims databases and reference standard data sources are often lacking due to restrictive contracts between data holders, data collection entities, and researchers. In addition, despite requiring considerable time and cost, validation studies on these topics—given that they are highly local and circumstances differ among countries—are rarely published in high-impact international journals, which may further discourage researchers from conducting such studies. In fact, only 36 validation studies were published in Japan between 2010 and 2022 (Yamana et al., 2023), resulting in the limited number of such studies available as references. Moreover, the PMDA guideline (Pharmaceuticals and Medical Devices Agency, 2020), requiring the calculation of positive predictive value and sensitivity, may also make it difficult for MAHs to conduct validation studies because calculating sensitivity can be challenging, especially when a gold standard is chart review. To address this situation, MAHs should consider collaborating with academia or encouraging academic research by offering funding opportunities. It could serve as an incentive for academia, which is also expected to further advance methodological development through increased collaborative research with industry. Additionally, its frequency has been decreasing, particularly after 2020. The issuance of the PMDA guideline in 2020, which clarified that only limited situations require validation, may have contributed to this trend. Further research on MAHs using interviews and qualitative research methods is required to completely understand the reason for the decline in the use of validated algorithms.

Several initiatives aimed at promoting validation studies are underway in Japan. Recently, some hospital data networks have enabled validation studies by overcoming the aforementioned barriers (Tanigawa et al., 2022; Yamana et al., 2017; Kubota et al., 2021). In addition, the 2023 amendment to the Next-Generation Medical Infrastructure Act links anonymized medical information data from hospitals to the National Database, allowing MAHs to use pseudonymized medical information for drug approval (Cabinet Office Government of Japan, 2020). These amendments promote validation studies in Japan, conducted by both academia and MAHs. In addition, the PMDA established the Consortium for the Promotion of Medical Information Databases in 2021 to discuss outcome definitions with MAHs and database holders (Pharmaceuticals and Medical Devices Agency, 2021). Some deliverables from the Consortium are published on its website, including a list of validation studies that meet the PMDA criteria, as well as a protocol template for validation studies. In addition, the Japanese Society of Pharmacoepidemiology, the Japan Epidemiological Association, and the Society of Clinical Epidemiology have established an Outcome Definition Repository that aggregates outcome algorithms used in previous Japanese pharmacoepidemiological studies and includes their validation information to share and reuse existing claims-based algorithms (Keiya et al., 2021). These initiatives are expected to promote validation studies and their results, thereby facilitating the use of validated claims-based algorithms in post-marketing database studies.

This study has some limitations. First, the data were based on information from the pharmacovigilance plan of J-RMPs. We did not examine whether validated claims-based algorithms were ultimately used in post-marketing database studies because few such studies have been completed since the introduction of the Good Post-Marketing Study Practice in 2018. In addition, it will also be important to examine whether quantitative bias analysis is conducted in post-marketing database studies. This method, which assesses the impact of potential misclassification bias on the estimated association, was recently recommended (International council for harmonisation of technical requirements for pharmaceuticals for human Use, 2024b). Further evaluation is needed when more studies are completed, and their final reports are published. Second, we could not investigate the detailed methods used in validation studies because not everything has been made public; prompt publication, with transparency and independent assessment (e.g., peer-review process), is expected. Third, the small sample size limits the robustness of subgroup analyses, such as effectiveness issues and ICD-10 classification. Therefore, caution should be exercised to avoid overinterpreting the results of subgroup analyses. Fourth, as this study had a data cut-off in 2023, recent data could not be included. Fifth, this study is a preliminary descriptive analysis conducted in one country and did not include in-depth comparisons with other countries because no systematic review of the use of validated claims-based algorithms in regulatory settings in other countries has been reported. Therefore, larger and international comparative studies are needed.

The frequency of validated algorithm use varied across issue types in this study, and was especially high in effectiveness issues, although the sample size was small. The overall use in safety assessment was limited, suggesting that the results from these post-marketing database studies may not provide sufficient evidence. This indicates the need for further promotion of validated claims-based algorithm use to enhance the level of evidence in post-marketing studies using claims data.

## Data Availability

The original contributions presented in the study are included in the article/Supplementary Material, further inquiries can be directed to the corresponding author.
