# Supplementary material for: Trends in the use of validated claims-based algorithms in Japanese post-marketing database studies
Source: Front Pharmacol. 2025 Sep 2;16:1642490. doi: 10.3389/fphar.2025.1642490 (PMC12436343; doi:10.3389/fphar.2025.1642490)
Supplement: Supplementary file 1 [file Supplementaryfile1.docx]

Supplementary Material

Supplemental Table 1. List of issues met exclusion criteria 1 ,2, or 3 (n=26)

| **No** | **Product name** | **Fiscal year*** | **Issue type** | **Issues** | **Database** | **Exclusion criteria** |
| --- | --- | --- | --- | --- | --- | --- |
| 1 | Ibrance | 2017 | IR | Interstitial lung disease | － | Database selection in progress |
| 2 | Arleda | 2019 | IR | Ischemic heart disease | － | Database selection in progress |
| 3 | Arleda | 2019 | PR | Fractures | － | Database selection in progress |
| 4 | Vyvespi | 2019 | IR | Cardiovascular events | － | Database selection in progress |
| 5 | Breztri | 2019 | IR | Cardiovascular events | － | Database selection in progress |
| 6 | Trintellix | 2019 | PR | Bleeding | － | Database selection in progress |
| 7 | Rinvoq | 2019 | IR | Serious infections (including tuberculosis, pneumonia, pneumocystis pneumonia, sepsis, opportunistic infections) | － | Database selection in progress |
| 8 | Rinvoq | 2019 | IR | Venous thromboembolism | － | Database selection in progress |
| 9 | Rinvoq | 2019 | PR | Cardiovascular events | － | Database selection in progress |
| 10 | Rinvoq | 2019 | PR | Malignancies | － | Database selection in progress |
| 11 | Latuda | 2019 | IR | Hyperglycemia/Diabetic ketoacidosis/Diabetic coma | － | Database selection in progress |
| 12 | Latuda | 2019 | PR | Hypoglycemia | － | Database selection in progress |
| 13 | Emgality | 2020 | MI | Serious cardiovascular events | － | Database selection in progress |
| 14 | Entresto | 2021 | IR | Hypotension | － | Database selection in progress |
| 15 | Entresto | 2021 | IR | Renal impairment/Renal failure | － | Database selection in progress |
| 16 | Entresto | 2021 | IR | Hyperkalemia | － | Database selection in progress |
| 17 | Adalimumab "MA" | 2021 | IR | Serious infections | － | Database selection in progress |
| 18 | Adalimumab "MA" | 2021 | IR | Serious tuberculosis | － | Database selection in progress |
| 19 | Moizerto | 2021 | IR | Skin infections | － | Database selection in progress |
| 20 | Reyvow | 2021 | MI | Use in pregnancy | － | Database selection in progress |
| 21 | Treprostinil | 2022 | IR | Hypotension/Syncope | － | Database selection in progress |
| 22 | Skyrizi | 2022 | IR | Serious infections | － | Database selection in progress |
| 23 | Epadel | 2022 | PR | Bleeding tendency | － | Database selection in progress |
| 24 | Spikevax | 2021 | Other specific risk | Specific adverse events observed in the acute phase after vaccination (patient reported outcome using app) | JMDC/Pep Up | Safety issue not listed in the safety specification in RMP |
| 25 | Spikevax | 2021 | Other specific risk | Serious events requiring hospitalization observed in the non-acute phase after vaccination | JMDC/Pep Up | Safety issue not listed in the safety specification in RMP |
| 26 | Lenvima | 2021 | IR | Hypothyroidism | MID-NET | Insufficiently documented |

*2017: 2017 April- 2018 March, 2018: 2018 April- 2019 March, 2019: 2019 April- 2020 March, 2020: 2020 April- 2021 March, 2021: 2021 April- 2022 March, 2022: 2022 April- 2023 January.

Supplemental Table 2. List of issues met exclusion criteria 4 (n=45)

| **No** | **Product name** | **Fiscal Year*** | **Issue type** | **Issues** | **ICD-10 Major Classification** | **Study element**** | **Database** | **Exclusion criteria 4 (data source for definition)** |
| --- | --- | --- | --- | --- | --- | --- | --- | --- |
| 1 | Viltepso | 2019 | EI | Efficacy on motor function, etc. | G | Outcome | Disease registry | Registry |
| 2 | Actemra | 2019 | EI | Efficacy of Actemra intravenous injection in patients with cytokine release syndrome associated with tumor-specific T-cell infusion therapy | D | Outcome/population | Disease registry | Registry |
| 3 | Nexviazyme | 2021 | EI | Effectiveness [Pompe disease] under actual use conditions | E | Outcome | Disease registry | Registry |
| 4 | Ibrance | 2017 | IR | Myelosuppression (neutropenia) | D | Outcome | MID-NET | Laboratory test results |
| 5 | Neirin | 2017 | IR | Hepatic impairment | K | Outcome | MID-NET | Laboratory test results |
| 6 | Pralia | 2017 | IR | Hypocalcemia | E | Outcome | MID-NET | Laboratory test results |
| 7 | Atozet | 2018 | IR | Hepatic dysfunction/Fulminant hepatitis/Hepatitis/Jaundice | K | Outcome | MID-NET | Laboratory test results |
| 8 | Atozet | 2018 | IR | Hyperglycemia/Diabetes | E | Outcome | MID-NET | Laboratory test results |
| 9 | Evenity | 2018 | IR | Hypocalcemia | E | Outcome | MID-NET | Laboratory test results |
| 10 | Minnebro | 2018 | IR | Hyperkalemia (appropriate use) | E | Outcome | MID-NET/MDV | Laboratory test results |
| 11 | Minnebro | 2018 | IR | Hyperkalemia (compared to control) | E | Outcome | MID-NET | Laboratory test results |
| 12 | Portrazza | 2019 | IR | Hypomagnesemia | E | Outcome | MDV | Laboratory test results |
| 13 | Bevacizumab "Pfizer" | 2019 | IR | Proteinuria/Nephrotic syndrome | N | Outcome | MDV | Laboratory test results |
| 14 | Bevacizumab "Pfizer" | 2019 | IR | Myelosuppression | D | Outcome | MDV | Laboratory test results |
| 15 | Rituximab "Pfizer" | 2019 | IR | Pancytopenia/Leukopenia/Neutropenia/Agranulocytosis/Thrombocytopenia | D | Outcome | MDV | Laboratory test results |
| 16 | Fycompa | 2019 | IR | Hostility and aggression | F | Outcome | Real-world data | Primary data collection only for outcome |
| 17 | Kerendia | 2021 | IR | Hyperkalemia | E | Outcome | MID-NET | Laboratory test results |
| 18 | Imbruvica | 2022 | IR | Bleeding | I | Outcome | Disease registry | Registry |
| 19 | Imbruvica | 2022 | IR | Myelosuppression | D | Outcome | Disease registry | Registry |
| 20 | Imbruvica | 2022 | IR | Infections | A | Outcome | Disease registry | Registry |
| 21 | Imbruvica | 2022 | IR | Arrhythmia | I | Outcome | Disease registry | Registry |
| 22 | Imbruvica | 2022 | IR | Hypersensitivity | T | Outcome | Disease registry | Registry |
| 23 | Imbruvica | 2022 | IR | Ocular disorders | H | Outcome | Disease registry | Registry |
| 24 | Imbruvica | 2022 | IR | Hepatic failure/Hepatic impairment | K | Outcome | Disease registry | Registry |
| 25 | Imbruvica | 2022 | IR | Interstitial lung disease | J | Outcome | Disease registry | Registry |
| 26 | Imbruvica | 2022 | IR | Secondary malignancies | C | Outcome | Disease registry | Registry |
| 27 | Imbruvica | 2022 | IR | Drug interactions with CYP3A inhibitors | drug interaction | Exposure | Disease registry | Registry |
| 28 | Imbruvica | 2022 | IR | Use in patients with hepatic impairment | hepatic impairment | Population | Disease registry | Registry |
| 29 | Atozet | 2018 | MI | Patients with hepatic impairment | hepatic impairment | Population | MID-NET | Laboratory test results |
| 30 | Evenity | 2018 | MI | Safety in patients with renal impairment　（indicaiton: Hypocalcemia） | renal impairment | Population | MID-NET | Laboratory test results |
| 31 | Evenity | 2018 | MI | Safety in patients with renal impairment　（indicaition:CV） | renal impairment | Population | MID-NET | Laboratory test results |
| 32 | Viltepso | 2019 | MI | Safety profile in long-term treated patients and patients with disease progression | long-term | long-term/population | Disease registry | Registry |
| 33 | Viltepso | 2019 | MI | Safety profile in patients with renal impairment | renal impairment | Population | Disease registry | Registry |
| 34 | Ibrance | 2017 | PR | Safety in patients with hepatic impairment | hepatic impairment | Population | MID-NET | Laboratory test results |
| 35 | Tecentriq | 2018 | PR | Hematologic toxicity (neutropenia, febrile neutropenia) when used in combination with chemotherapy | D | Outcome/Exposure | MID-NET | Laboratory test results |
| 36 | Urece | 2019 | PR | Hepatic impairment | K | Outcome | MID-NET | Laboratory test results |
| 37 | Lokelma | 2019 | PR | Hypokalemia | E | Outcome | MID-NET/MDV | Laboratory test results |
| 38 | Viltepso | 2019 | PR | Hypersensitivity | T | Outcome | Disease registry | Registry |
| 39 | Viltepso | 2019 | PR | Renal impairment | N | Outcome | Disease registry | Registry |
| 40 | Viltepso | 2019 | PR | Transitional cell carcinoma of the urinary tract/Effects on the urinary system | N | Outcome | Disease registry | Registry |
| 41 | Kerendia | 2021 | PR | Renal impairment | N | Outcome | MID-NET | Laboratory test results |
| 42 | Imbruvica | 2022 | PR | Stevens-Johnson syndrome | L | Outcome | Disease registry | Registry |
| 43 | Xarelto | 2022 | MI | Safety in patients with renal impairment (pediatric VTE) | renal impairment | Population | MDV | Laboratory test results |
| 44 | Xarelto | 2022 | MI | Safety in patients with renal impairment (PAD after revascularization) | renal impairment | Population | MDV | Laboratory test results |
| 45 | Xarelto | 2022 | MI | Safety in low body weight adults (PAD after revascularization) | low body weight | Population | MDV | Laboratory test results |

*2017: 2017 April- 2018 March, 2018: 2018 April- 2019 March, 2019: 2019 April- 2020 March, 2020: 2020 April- 2021 March, 2021: 2021 April- 2022 March, 2022: 2022 April- 2023 January. ** When the main content of an issue covered more than one study element, the primary element (i.e., the first described) was used for statistical analysis.

Supplemental Table 3-1. Effectiveness issues (n=3)

| Issue type | No | Product name | Fiscal Year* | Issues | ICD-10 Major Classification | Study element** | Database | Validated claims based algorithm use*** |
| --- | --- | --- | --- | --- | --- | --- | --- | --- |
| Effectiveness | 1 | Spiramycin | 2018 | Effectiveness [Prevention of fetal infection] under real-world usage conditions | Q | Outcome | JMDC | 1 |
| Effectiveness | 2 | Efient | 2021 | Effectivenessin [Ischemic heart disease, Prevention of recurrence after ischemic cerebrovascular disease] patients with atherothrombotic or lacunar infarction at high risk of recurrence | I | Outcome/  population | MID-NET/JMDC | 2 |
| Effectiveness | 3 | Parmodia | 2021 | Effectiveness on prevention of cardiovascular events | I | Outcome | MID-NET | 2 |

*2017: 2017 April- 2018 March, 2018: 2018 April- 2019 March, 2019: 2019 April- 2020 March, 2020: 2020 April- 2021 March, 2021: 2021 April- 2022 March, 2022: 2022 April- 2023 January. **When the main content of an issue covered more than one study element, the primary element (i.e., the first described) was used for statistical analysis. ***0 = No description of a plan to use a validated claims-based algorithm, 1 = Description of a plan to use a validated claims-based algorithm by conducting a new validation study, 2 = Description of a plan to use a validated claims-based algorithm by referring to an existing validation study

Supplemental Table 3-2. Important Identified Risk (n=47)

| **Issue type** | **No** | **Product name** | **Fiscal Year*** | **Issues** | **ICD-10 Major Classification** | **Study element** | **Database** | **Validated claims-based algorithm use**** |
| --- | --- | --- | --- | --- | --- | --- | --- | --- |
| IR | 1 | Olumiant | 2017 | Serious infections (including tuberculosis, pneumonia, etc.) | A | Outcome | MDV | 1 |
| IR | 2 | Rexulti | 2017 | Hyperglycemia / Diabetic ketoacidosis / Diabetic coma | E | Outcome | MID-NET | 0 |
| IR | 3 | Rexulti | 2017 | Agranulocytosis / Leukopenia | D | Outcome | MID-NET | 1 |
| IR | 4 | Atozet | 2018 | Rhabdomyolysis / Myopathy | M | Outcome | MID-NET | 0 |
| IR | 5 | Sivextro | 2018 | Myelosuppression (thrombocytopenia, decreased hemoglobin, neutropenia, etc.) | D | Outcome | MDV | 1 |
| IR | 6 | Sivextro | 2018 | Peripheral neuropathy and optic neuritis | G | Outcome | MDV | 1 |
| IR | 7 | Sivextro | 2018 | Lactic acidosis | E | Outcome | MDV | 1 |
| IR | 8 | Verzenio | 2018 | Interstitial lung disease | J | Outcome | MDV | 0 |
| IR | 9 | Trastuzumab (Daiichi Sankyo) | 2018 | Cardiac disorder | I | Outcome | MDV | 0 |
| IR | 10 | Trastuzumab (Daiichi Sankyo) | 2018 | Infusion reaction | T | Outcome | MDV | 0 |
| IR | 11 | Trastuzumab (Daiichi Sankyo) | 2018 | Interstitial pneumonia / Lung disorder | J | Outcome | MDV | 0 |
| IR | 12 | Trastuzumab (Daiichi Sankyo) | 2018 | Hematologic toxicity | D | Outcome | MDV | 0 |
| IR | 13 | Trastuzumab (Daiichi Sankyo) | 2018 | Hepatic failure / Hepatic disorder | K | Outcome | MDV | 0 |
| IR | 14 | Trastuzumab (Daiichi Sankyo) | 2018 | Coma / Cerebrovascular disorder / Cerebral edema | I | Outcome | MDV | 0 |
| IR | 15 | Trastuzumab (Daiichi Sankyo) | 2018 | Renal disorder | N | Outcome | MDV | 0 |
| IR | 16 | Trastuzumab (Daiichi Sankyo) | 2018 | Infections | A | Outcome | MDV | 0 |
| IR | 17 | Suglat | 2018 | Hypoglycemia | E | Outcome | JMDC | 0 |
| IR | 18 | Suglat | 2018 | Effects due to ketoacidosis / increased ketone bodies | E | Outcome | JMDC | 0 |
| IR | 18 | Infliximab (Pfizer) | 2018 | Serious infections | A | Outcome | MDV | 0 |
| IR | 20 | Infliximab (Pfizer) | 2018 | Tuberculosis | A | Outcome | MDV | 0 |
| IR | 21 | Infliximab (Pfizer) | 2018 | Serious hematologic disorders | D | Outcome | MDV | 0 |
| IR | 22 | Infliximab (Pfizer) | 2018 | Interstitial pneumonia | J | Outcome | MDV | 0 |
| IR | 23 | Trelegy | 2018 | Pneumonia | J | Outcome | MDV | 0 |
| IR | 24 | Forxiga | 2019 | Effects of increased ketone bodies / Ketoacidosis | E | Outcome | JMDC | 0 |
| IR | 25 | Smyraf | 2019 | Serious infections (including tuberculosis, pneumonia, etc.) | A | Outcome | MDV | 2 |
| IR | 26 | Portrazza | 2019 | Arterial thromboembolism | I | Outcome | MDV | 0 |
| IR | 27 | Portrazza | 2019 | Venous thromboembolism | I | Outcome | MDV | 0 |
| IR | 28 | Fiasp | 2019 | Hypoglycemia | E | Outcome | MDV | 0 |
| IR | 29 | Bevacizumab (Daiichi Sankyo) | 2019 | Shock / Anaphylaxis / Hypersensitivity / Infusion reaction | T | Outcome | MDV | 1 |
| IR | 30 | Bevacizumab (Pfizer) | 2019 | Bleeding | I | Outcome | MDV | 0 |
| IR | 31 | Bevacizumab (Pfizer) | 2019 | Hypertension / Hypertensive crisis | I | Outcome | MDV | 0 |
| IR | 32 | Rituximab (Pfizer) | 2019 | Infections | A | Outcome | MDV | 1 |
| IR | 33 | Lyumjev | 2019 | Severe hypoglycemia | E | Outcome | MDV | 0 |
| IR | 34 | Lokelma | 2019 | Congestive heart failure | I | Outcome | MID-NET/MDV | 0 |
| IR | 35 | Soliqua | 2019 | Hypoglycemia | E | Outcome | MDV | 0 |
| IR | 36 | Atecurra | 2020 | Cardiovascular events | I | Outcome | MDV | 0 |
| IR | 37 | Enazea | 2020 | Cardiovascular events | I | Outcome | MDV | 0 |
| IR | 38 | Adalimumab (FKB) | 2020 | Serious infections | A | Outcome | JMDC | 0 |
| IR | 39 | Spikevax | 2021 | Shock, anaphylaxis | T | Outcome | JMDC | 0 |
| IR | 40 | Binzellex | 2021 | Serious infections | A | Outcome | MDV | 0 |
| IR | 41 | Treprost | 2022 | Bleeding | I | Outcome | MID-NET/JMDC | 0 |
| IR | 42 | Xarelto | 2022 | Bleeding (pediatric VTE) | I | Outcome | MDV | 0 |
| IR | 43 | Xarelto | 2022 | Bleeding (PAD after revascularization) | I | Outcome | MDV | 0 |
| IR | 44 | Xarelto | 2022 | Concomitant use with antiplatelet and NSAIDs (pediatric VTE) | drug interaction | Exposure | MDV | 0 |
| IR | 45 | Xarelto | 2022 | Concomitant use with antiplatelet and NSAIDs (PAD after revascularization) | drug interaction | Exposure | MDV | 0 |
| IR | 46 | Sotyktu | 2022 | Serious infections | A | Outcome | MDV | 0 |
| IR | 47 | Bevacizumab (CTNK) | 2022 | Venous thromboembolism | I | Outcome | MDV | 0 |

*2017: 2017 April- 2018 March, 2018: 2018 April- 2019 March, 2019: 2019 April- 2020 March, 2020: 2020 April- 2021 March, 2021: 2021 April- 2022 March, 2022: 2022 April- 2023 January. **0 = No description of a plan to use a validated claims-based algorithm, 1 = Description of a plan to use a validated claims-based algorithm by conducting a new validation study, 2 = Description of a plan to use a validated claims-based algorithm by referring to an existing validation study

Supplemental Table 3-3. Important potential risks (n=13)

| **Issue type** | **No** | **Product name** | **Fiscal Year*** | **Issues** | **ICD-10 Major Classification** | **Study element** | **Database** | **Validated claims-based algorithm use***** |
| --- | --- | --- | --- | --- | --- | --- | --- | --- |
| PR | 1 | Olumiant | 2017 | Malignant neoplasms | C | Outcome | MDV | 1 |
| PR | 2 | Rexulti | 2017 | Hypoglycemia | E | Outcome | MID-NET | 1 |
| PR | 3 | Evenity | 2018 | Serious cardiovascular events | I | Outcome | MID-NET | 0 |
| PR | 4 | Tecentriq | 2018 | febrile neutropenia with chemotherapy | D | Outcome | MDV | 0 |
| PR | 5 | Tecentriq | 2018 | Infections with chemotherapy | A | Outcome | MDV | 0 |
| PR | 6 | Infliximab (Pfizer) | 2018 | Malignant neoplasms | C | Outcome | MDV | 0 |
| PR | 7 | Tarlige | 2018 | Heart failure / pulmonary edema | I | Outcome | MID-NET | 2 |
| PR | 8 | Smyraf | 2019 | Malignant neoplasms | C | Outcome | MDV | 2 |
| PR | 9 | Binzellex | 2021 | Malignant neoplasms | C | Outcome | MDV | 0 |
| PR | 10 | Xarelto | 2022 | Concomitant use with CYP3A4 inhibitors (pediatric VTE) | Exposure: drug interaction | Exposure | MDV | 0 |
| PR | 11 | Xarelto | 2022 | Concomitant use with CYP3A4 inhibitors (PAD after revascularization) | Exposure: drug interaction | Exposure | MDV | 0 |
| PR | 12 | Sotyktu | 2022 | Malignant neoplasms | C | Outcome | MDV | 0 |
| PR | 13 | Ostabalo | 2022 | Cardiovascular events | I | Outcome | MDV / Planning** | 0 |

*2017: 2017 April- 2018 March, 2018: 2018 April- 2019 March, 2019: 2019 April- 2020 March, 2020: 2020 April- 2021 March, 2021: 2021 April- 2022 March, 2022: 2022 April- 2023 January. **Only MDV was considered in the analysis for DB. ***0 = No description of a plan to use a validated claims-based algorithm, 1 = Description of a plan to use a validated claims-based algorithm by conducting a new validation study, 2 = Description of a plan to use a validated claims-based algorithm by referring to an existing validation study

Supplemental Table 3-4. Important missing information (n=5)

| **Issue type** | **No** | **Product name** | **Fiscal Year*** | **Issues** | **ICD-10 major classification** | **Study element** | **Database** | **Validated claims-based algorithm use***** |
| --- | --- | --- | --- | --- | --- | --- | --- | --- |
| MI | 1 | Zoltofi | 2019 | Safety when switching from GLP-1 receptor agonists (including in combination with insulin) to this drug | drug switching | Exposure | MDV | 0 |
| MI | 2 | Soliqua | 2019 | Safety when switching from GLP-1 receptor agonists (including in combination with insulin) to this combination drug | drug switching | Exposure | MDV / Planning** | 0 |
| MI | 3 | Xarelto | 2022 | Safety in patients who received other oral anticoagulants prior to this drug (pediatric VTE) | drug switching | Exposure | MDV | 0 |
| MI | 4 | Xarelto | 2022 | Safety with long-term administration (pediatric VTE) | long-term use | Exposure | MDV | 0 |
| MI | 5 | Xarelto | 2022 | Safety with long-term administration (PAD after revascularization) | long-term use | Exposure | MDV | 0 |

*2017: 2017 April- 2018 March, 2018: 2018 April- 2019 March, 2019: 2019 April- 2020 March, 2020: 2020 April- 2021 March, 2021: 2021 April- 2022 March, 2022: 2022 April- 2023 January. ** Only MDV was considered in the analysis for DB. ***Only MDV was considered in the analysis for DB. ***0 = No description of a plan to use a validated claims-based algorithm, 1 = Description of a plan to use a validated claims-based algorithm by conducting a new validation study, 2 = Description of a plan to use a validated claims-based algorithm by referring to an existing validation study

Supplemental Table 4. List of issues for which algorithms validated in existing validation studies are planned to be used

| **Issue type** | **Product name** | **Issues** | **Database** | **Excerpt from a description in RMPs regarding the reference to existing validation studies** | **Notes** |
| --- | --- | --- | --- | --- | --- |
| Effectiveness | Efient | Effectiveness in [Ischemic heart disease, Prevention of recurrence after ischemic cerebrovascular disease] patients with atherothrombotic or lacunar infarction at high risk of recurrence | MID-NET/JMDC | Use the outcome definition of “acute ischemic stroke treated during hospitalization” that has already been validated in MID-NET. | The validation study referenced here is listed on the PMDA website as a study conducted by the PMDA using MID-NET.  <https://www.pmda.go.jp/files/000225081.pdf>  Tanigawa M, et al. Pharmacoepidemiol Drug Saf. 2022;31(5):524-533.   - - Gold standard: medical chart review   - Validity index: PPV (62%-90.9%) and sensitivity (33%-100%) |
| Effectiveness | Parmodia | Effectiveness on prevention of cardiovascular events | MID-NET | The details of the outcome definition will be considered based on discussions during the epidemiological study planning consultation and the validation results from MID-NET. | The validation study referenced here is listed on the PMDA website as a study conducted by the PMDA using MID-NET.  <https://www.pmda.go.jp/files/000225081.pdf>  Tanigawa M, et al. Pharmacoepidemiol Drug Saf. 2022;31(5):524-533.   - - Gold standard: medical chart review   - Validity index: PPV (62%-90.9%) and sensitivity (33%-100%) |
| IR | Smyraf | Serious infections (including tuberculosis, pneumonia, etc.) | MDV | The results of a validation study being conducted by Medical Data Vision (MDV) Co., Ltd. to examine the validity of the outcome definition in the MDV database will be used. | Nishikawa A, et al. Ann Clin Epidemiol. 2022;4(1):20-31.   - - Gold standard: medical chart review   - Validity indices: PPV (85.6%) and pseudo sensitivity (100%) of the most recommended algorithm |
| PR | Smyraf | Malignant neoplasms | MDV | The results of a validation study being conducted by Medical Data Vision (MDV) Co., Ltd. to examine the validity of the outcome definition in the MDV database will be used. | Nishikawa A, et al. Ann Clin Epidemiol. 2022;4(1):20-31.   - - Gold standard: medical chart review   - Validity indices: PPV (90.7%) and pseudo sensitivity (64.1%) of the most recommended algorithm |
| PR | Tarlige | Heart failure / pulmonary edema | MID-NET | A validation study of the outcome definition for heart failure has been conducted in MID-NET®. Therefore, it was determined that a post-marketing database study would be conducted using MID-NET®. | The validation study referenced here is listed on the PMDA website as a study conducted by the PMDA using MID-NET.  <https://www.pmda.go.jp/files/000225081.pdf>  This validation study has not been published yet, but the results of the validation study are disclosed only to MID-NET users. |

PMDA: Pharmaceuticals and Medical Devices Agency (Japanese pharmaceutical regulatory authority)
